# Supplementary material for: Multi-mode excitation drives disorder during the ultrafast melting of a C4-symmetry-broken phase
Source: Nat Commun. 2022 Jan 11;13:238. doi: 10.1038/s41467-021-27819-y (PMC8752725; doi:10.1038/s41467-021-27819-y)
Supplement: Supplementary file 1 — Supplementary Information [file 41467_2021_27819_MOESM1_ESM.pdf]

Supplementary Information: Multi-mode excitation drives disorder during the ultrafast melting of a C4-symmetry-broken phase

Daniel Perez-Salinas[1*], Allan S. Johnson[1*], D. Prabhakaran[2], Simon Wall[1,3]

[1]*ICFO – The Institute of Photonics Sciences, The Barcelona Institute of Science and Technology, 08860, Castelldefels, Barcelona, Spain*

[2]*Department of Physics, Clarendon Laboratory, University of Oxford, Oxford OX1 3PU, United Kingdom*

[3]*Department of Physics and Astronomy, Aarhus University, Ny Munkegade 120, 8000 Aarhus C, Denmark*

**Equal contribution*

Supplementary Methods

S1 Ultrafast reflection anisotropy setup

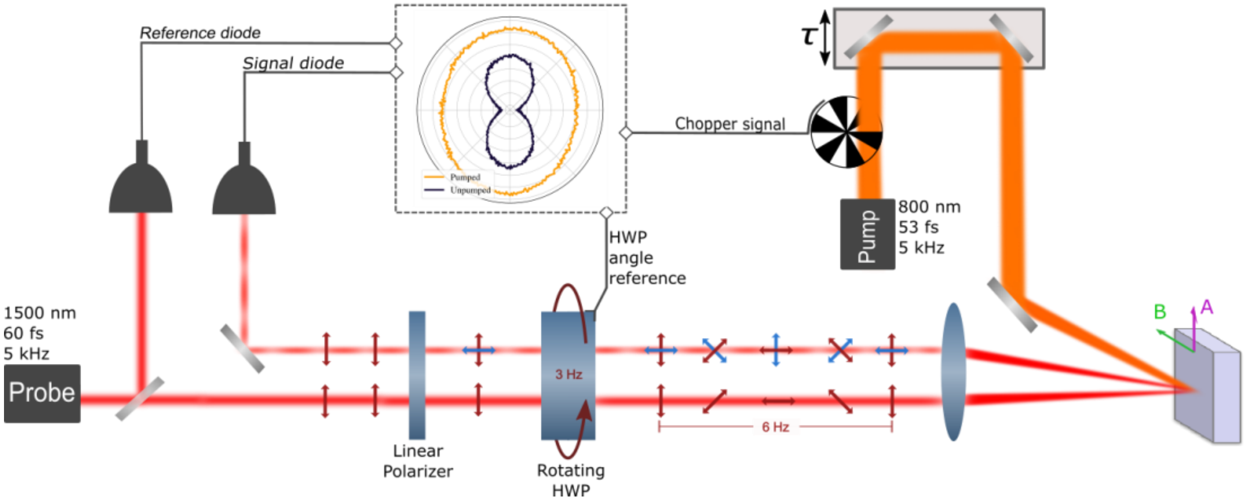

Supplementary Figure S1: Schematic of the reflection anisotropy setup. To measure the reflection anisotropy, we adopt a double-pass scheme using an optical parametric amplifier (OPA) operating at 5 kHz. A fraction of the output of the OPA is first sampled using a beam splitter and sent to a reference diode to normalize intensity fluctuations. The rest of the beam passes through a fixed wire-grid linear polarizer to define the polarization state, and then passes through a rotating waveplate (3Hz) which

causes a rotation of the polarization by θ with respect to the initial polarization state. The beam is then focused onto the sample with a lens (f = 500 mm) though a 2 mm thick quartz cryostat window. The sample, mounted at near-normal incidence at the focal point of the lens, reflects the beam with a perturbed polarization state. The reflected beam passes back through the wave plate, causing the polarization to rotate by $-\theta$, undoing the initial rotation up to factor introduced by the sample (the rotation of the waveplate during the time taken to reflect from the sample is negligible). Finally, the light passes through the initial polarizer, transmitting only the component parallel to the incident light. The transmitted component is collected with an InGaAs photodiode. The duration of the probe beam, measured at the sample plane using frequency resolved optical gating, was approximately 60 fs (Supplementary Figure S2), and the focal spot diameter was 150 µm.

The sample was excited with an 800 nm pump pulse from the same laser, mechanically delayed with respect to the probe pulses, and modulated with an optical chopper at 2.5 kHz. The polarization was approximately aligned to the low reflectivity principal axis and the pulse duration was 50 fs (Supplementary Figure S2). The pump spot size at the sample was approximately 7.5 times that of the probe.

The sample and reference diodes were read by a four-channel oscilloscope along with the angle of the rotation stage and the chopper state on a shot-by-shot basis. The data was then filtered by angle and chopper state into separate on and off channels. As the rotation stage was not synchronized to the laser, the accuracy of the waveplate position is 0.5 degrees. Static measurements are performed in the same way, but the chopper signal is discarded.

S2 Pulse duration measurements

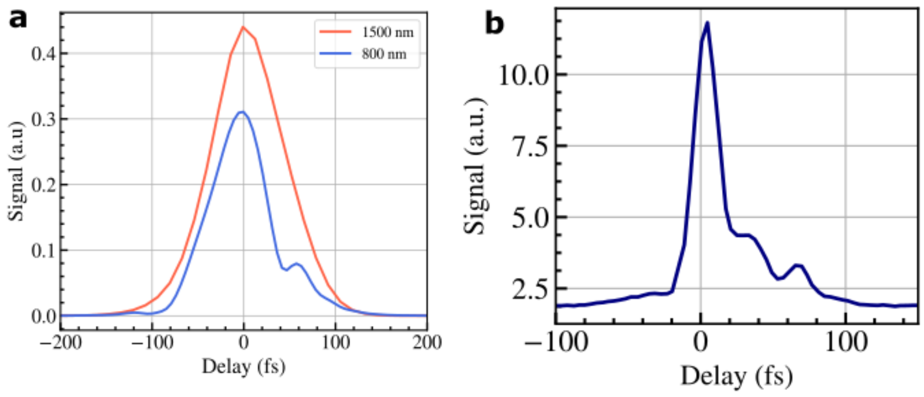

Supplementary Figure S2: Pump and probe pulse durations. **a** Pump and probe pulses used in the reflection anisotropy setup reconstructed using frequency-resolved optical gating[1]. The 800 nm pulses are of 50 fs duration (full-width half maximum) and the 1500 nm probe pulses are 60 fs duration. **b** Cross correlation of the 1800 nm pump and 650 nm probe pulses used in the high time resolution setup, measured in a 15 µm thick barium borate crystal. A full-width half maximum of 25 fs is obtained.

S3 Reflectivity of an anisotropic sample

We define the ab plane reflection coefficient as

$$R = \begin{pmatrix} r_1 e^{i\phi_1} & 0 \\ 0 & r_2 e^{i\phi_2} \end{pmatrix} = \alpha \begin{pmatrix} 1 & 0 \\ 0 & \rho e^{i\chi} \end{pmatrix},$$

where $\alpha = r_1 e^{i\phi_1}, \rho = \frac{r_2}{r_1}$, $\chi = \phi_2 - \phi_1$. For an arbitrary polarization state the reflectivity can be written as

$$R = \alpha \begin{pmatrix} \cos^2\theta + \rho e^{i\chi}\sin^2\theta & \frac{1}{2}\sin 2\theta\left(1 - \rho e^{i\chi}\right) \\ \frac{1}{2}\sin 2\theta\left(1 - \rho e^{i\chi}\right) & \sin^2\theta + \rho e^{i\chi}\cos^2\theta \end{pmatrix},$$

where θ is the angle made by the polarization relative to the crystal axis. Defining the input polarization as $\begin{pmatrix} 1 \\ 0 \end{pmatrix}$ and measuring the reflected parallel compoent, the detected intensity varies as

$$I_{VV} = \frac{r_1^2}{8}\left(4(1-\rho^2)\cos 2\theta + (1+\rho^2 - 2\rho\cos\chi)\cos 4\theta + 6 + 2\rho\cos\chi\right)$$

$$= I_{DC} + I_4 \cos 4\Phi + I_8 \cos 8\Phi.$$

Here $\Phi = \frac{\theta}{2}$ is the angle of the waveplate, which rotates the polarization at twice the rate of the waveplate rotation. $I_{DC}, I_4$and I_8 can be directly extracted from our data. All terms are sensitive to the birefringence ratio ρ, and thus the order parameter, as well as other factors.

We are unable to detect an eight-fold peak in our data; thus by setting $I_8 = 0$ we have $I_{DC} = \frac{r_1^2}{8}(7 + \rho^2)$ and $I_4 = \frac{r_1^2}{2}(1 - \rho^2)$. This allows us to solve for r_1 and ρ:

$$r_1^2 = I_{DC} + \frac{I_4}{4},$$

$$\rho^2 = \frac{4 - 7\frac{I_4}{I_{DC}}}{\left(4 + \frac{I_4}{I_{DC}}\right)}.$$

When the system is isotropic, $I_4 = 0$, which implies $\rho = 1$. Values of $\rho > 1$ correspond to domains of one parity, whereas $\rho < 1$ corresponds to domains of the alternative parity. Optical measurements cannot distinguish between domains of the same parity, therefore $\eta^2 \propto r_1 - r_2$.

We normalize η^2 to lie in the range ± 1 with

$$\eta^2 = 2\,\frac{r_1 - r_2}{r_1 + r_2} = \frac{1 - \rho}{1 + \rho}.$$

When fitting the data, we fit as a function of the waveplate angle using the following equation for I_{VV}:

$$I_{VV}^f = I_{DC} + I_2\cos(\theta + \psi_2) + I_4\cos(2\theta + \psi_4).$$

The I_2 term captures a small constant which is modulated at the frequency of the rotating waveplate. Such a term most likely corresponds to scatter from the waveplate to the detector. In addition, the

phase term ψ_4 allows us to correct for the fact that the crystal axis are not perfectly aligned to the polarization axis. It also allows us to observe the direction of strain, which is not confined along a particular direction.

S4 Pump color comparison

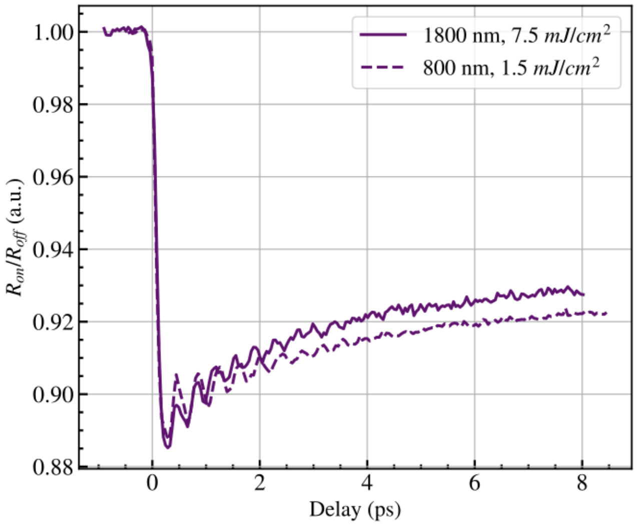

Supplementary Figure S3: Comparison of different pump wavelengths. Ultrafast reflection anisotropy measurements (as shown in Figure 2 and Figure 3a and b) were taken with 800 nm pump pulses, in contrast with the 1800 nm excitation used in the high time resolution setup (as shown in Figure 4c). To ensure the consistency of these two data sets we directly compare transient reflectivity dynamics measured with 800 nm and 1800 nm pump pulses in the high time resolution setup under identical probing condition. No appreciable differences are found, confirming the validity of the comparison.

S5 Probe color comparison

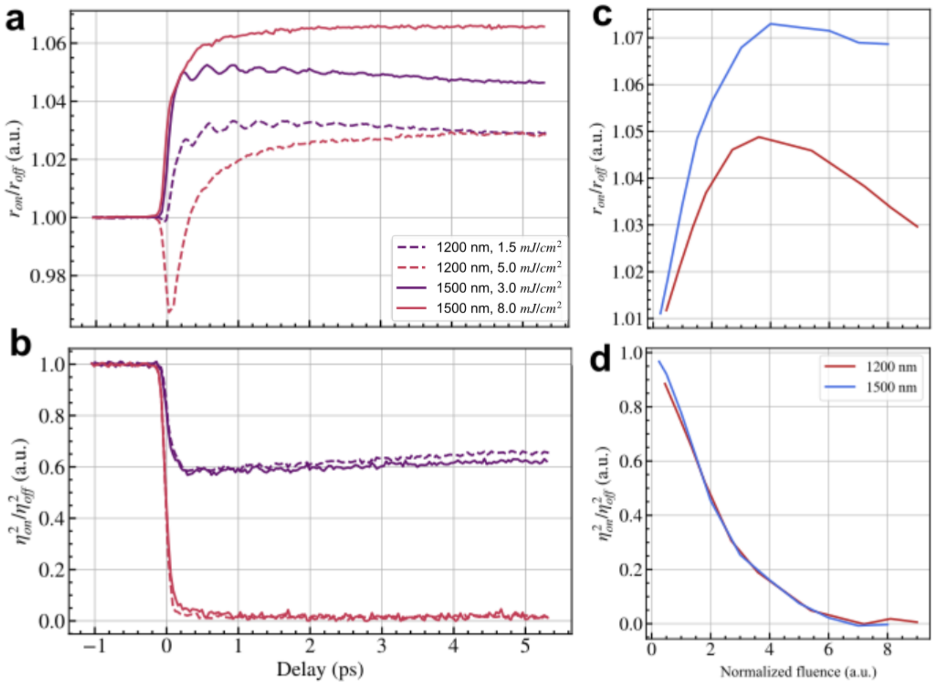

Supplementary Figure S4: Probe wavelength dependence of the ultrafast evolution of the isotropic and anisotropic reflectivity of LSMO. a Ultrafast evolution of the isotropic reflectivity r and **b** normalized order parameter η^2 probed with 1500 nm (continuous lines) and 1200 nm (dashed lines) pulses. The baseline non-zero value for η^2 induced by strain has been compensated through renormalization for both probe colors. Dynamics of the r parameter show strong wavelength dependence, however this variation is absent in the order parameter dynamics. **c** and **d** show the fluence dependence of the 1 ps delay value of r and η^2, respectively. The fluence axis for the 1200 nm data has been multiplied by 1.8 to account for the different penetration depths for the two probe colors, see following section.

S6 High temporal resolution data

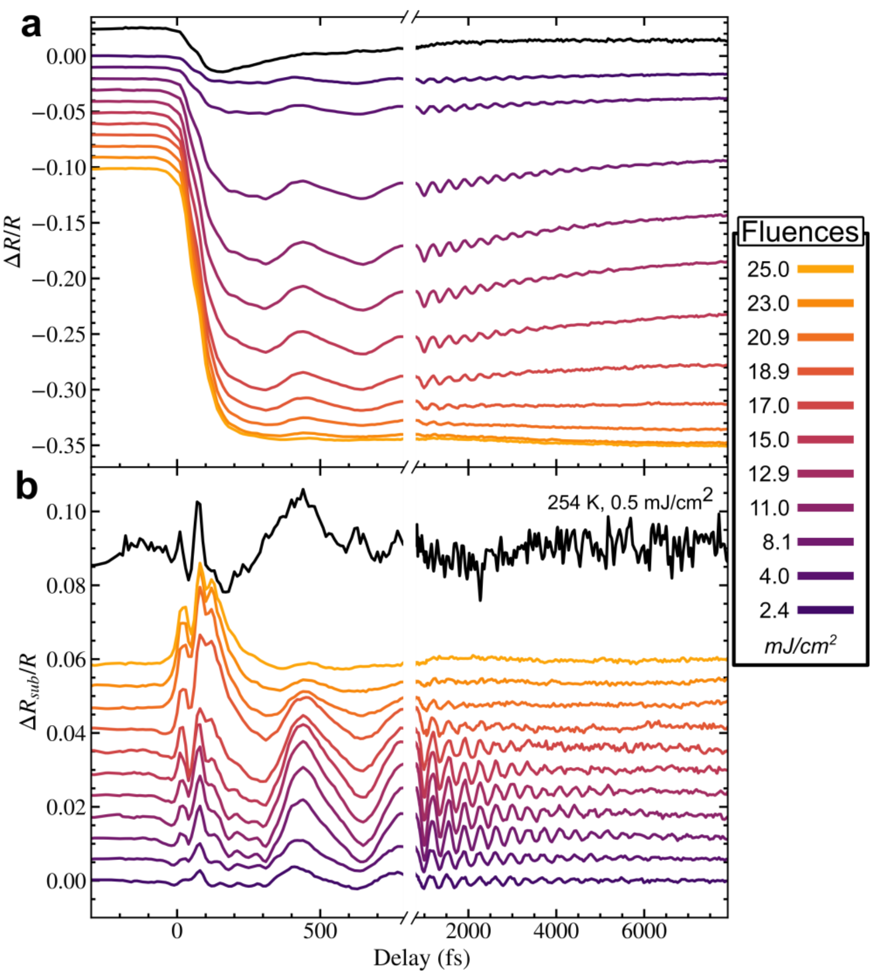

Supplementary Figure S5: Few-cycle 1800 nm Pump, 650 nm probe transient reflectivity data. Fluence dependent data (coloured) is performed at 140 K, well below T_{co}, while the black trace (T = 254 K, 0.5 mJ cm^{-2}) is performed above T_{co}. **a** Raw transient reflectivity changes (offset for clarity) and **b** background subtracted changes after fitting the data with multiple exponential decays. Measurements were performed at a near-normal angle of incidence as described in the methods section. Due to the different base temperatures and pump wavelengths, the fluences here are not directly comparable to the fluences used in the reflection anisotropy measurements in which the pump wavelength was 800 nm. With the exception of the absolute value of the threshold fluence, there was no significant difference in the dynamics with different pump wavelength (Supplementary Figure S3). At 140 K, we found that the sample could often, but not always, be switched to a meta-stable state with high fluence excitation. This results in a response that has different dynamics to the pristine state. Repeat measurements were performed to ensure that no meta-stable state was created in this case.

Supplementary Discussion

S7 Fluence dependence of the phase transition

We start by defining the local order parameter as the value of the order parameter at smallest valid length scale. For a dimerization transition, for example, this would be the degree of dimerization of the low temperature unit cell.

An initially inhomogenous 1st order phase transition

As LSMO exhibits a first order phase transition with surface melting, we first model the phase transition in which the local Tc is reduced as the surface is approached as

$$T_c(x) = T_B(1 - \frac{1}{(1+ax)^2})$$

Where x=0 corresponds to the surface of the crystal, and a is a constant that determines the thickness of the surface layer and T_B is the transition temperature of the bulk. We assume regions of the sample in which the temperature is below the local Tc are fully ordered ($\eta^2 = 1$), whereas the regions that are above the local Tc have no order ($\eta^2 = 0$). As a function of temperature, the system first starts to melt at the surface, with the ordered surface moving deeper into the bulk until all of the solid is transformed.

The probe light averages over a large length scale, penetrating exponentially into the material over a lengthscale l_{probe}. We assume that the measured order parameter is proportional to the intensity weighted average, i.e.

$$\langle \eta^2 \rangle = \frac{\int \eta^2(x) e^{-\frac{x}{l_{probe}}}}{\int e^{-\frac{x}{l_{probe}}}}$$

The average order parameter observed is shown in **Fig S6**.

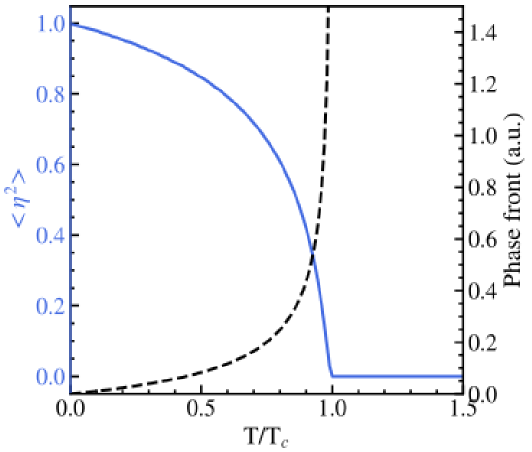

Supplementary Figure S6: Temperature dependence of the inhomogenous order parameter model. Dashed line corresponds to the position of the ordered surface relative to the crystal surface.

Pump-induced changes are then assumed to locally change the transition temperature additively. The temperature rise is dependent on the incident fluence, which decays exponentially from the surface. We assume that both phases absorb light equally. We consider three models for translating the absorbed fluence into a temperature rise, a constant heat capacity, a linear electronic heat capacity and a cubic heat capacity, corresponding to the low temperature limit of the Debye-Waller model.

Supplementary Figure S7 shows the results of such models for two different probe penetration depths. No significant difference is found. The main difference results from the fact that the same applied fluence results in different temperature jumps, but the overall shape is similar. In all cases the main observation, that the shorter penetrating probe reaches saturation sooner, is the same. These results were also robust to changes in the spatial dependence of T_c and the main requirement is that the surface must already show melting. When the phase front is linear with fluence, the data can be scaled to compensate for the penetration depth difference as shown in Supplementary Figure S4d.

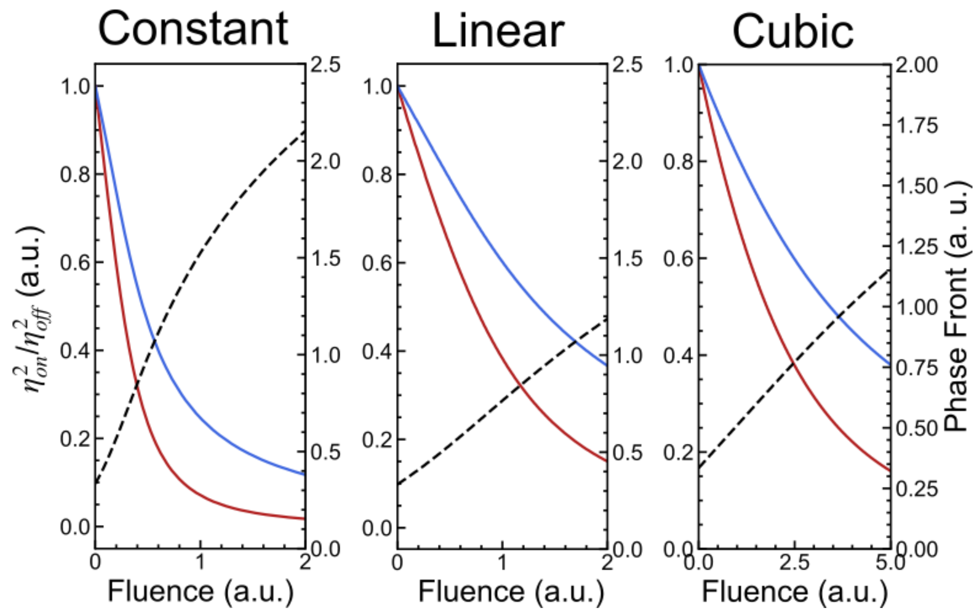

Supplementary Figure S7: Modelling the spatial dependence of the local temperature. We consider three cases. The case of a constant heat capacity, the temperature rise is linear with fluence $T(x,F) = T_0 + aFe^{-x/l_{pump}}$. The case of a heat capacity that is linear in temperature (an electronic heat capacity) the temperature rise follows a square root dependence $T(x,F) = T_0\sqrt{1 + bFe^{-x/l_{pump}}}$. Finally we also consider a heat capacity that depends cubically with temperature, corresponding to the low temperature limit of the Debye-Waller model, in which case the temperature rise is $T(x,F) = T_0(1 + cFe^{-x/l_{pump}})^{\frac{1}{4}}$

An initially homogenous 2nd order phase transition

For comparison, we use a homogenous second order model that has been previously used to characterize dynamical phase transitions. In this case, the value of the order parameter, locally, is initially independent of position in the crystal. The smooth transition results from the fact that the local (and global) order parameter size varies as $\eta^2 \propto |T - T_c|^p, T < T_c$. The average order parameter is measured in calculated in the same way as the inhomogenous first-order case and the temperature

dependence is shown in Supplementary Figure S8a. The value of p is chosen (p=0.2) to resemble the inhomogenous first-order case, but the following results are qualitatively similar for reasonable values of p around this value (Supplementary Figure S9).

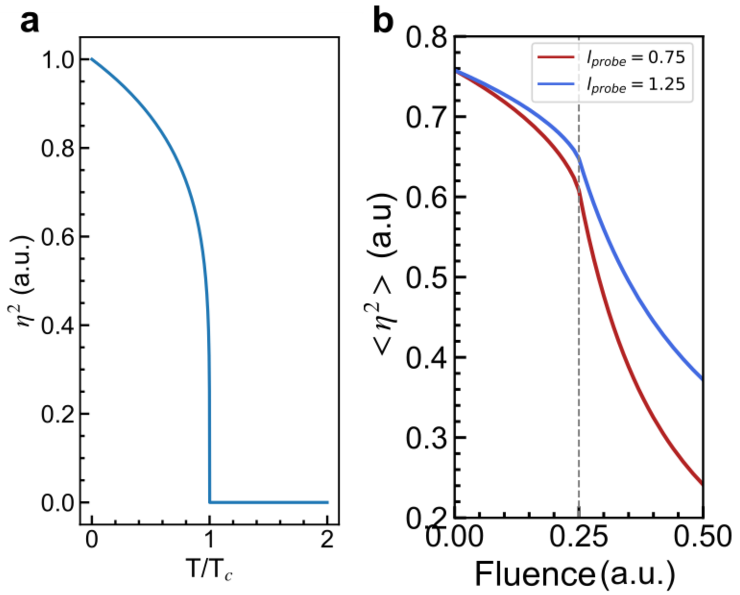

Supplementary Figure S8: Modelling a homogeneous 2nd order phase transition. **a** Temperature dependence of the order parameter in the homogeneous 2nd order transition model. **b** Sampled order parameter as a function of fluence for probes with different penetration depth. Dashed line indicates where part of the sample first reaches T_c.

We then change the local temperature in the same way as for the inhomogenous model. As for the homogenous model, the results were qualitatively the same for different models of the heat capacity, thus we only show the case of a constant heat capacity. The resulting fluence dependence is shown in Supplementary Figure S8b. An initial decrease is observed in which the order parameter at the surface is partially suppressed. At a critical fluence a strong discontinuity is observed which corresponds to the first time part of the sample goes above T_c. As the hottest point is always the surface of the crystal. This point is seen at the same fluence independent of the probe wavelength. The probe wavelength only defines the magnitude of the effect, with more surface sensitive colours showing a larger change. This kink is not observed in the experimental data shown in Figure 4d. However, as shown in Supplementary Figure S9, the contrast of this kink is reduced progressively as the value of p increases towards 1. Higher signal-to-noise may be required to observe the kink if the transition was described by a critical exponent significantly higher than the thermal one.

The value of the critical exponent does, however, strongly affect the temperature dependence of the order parameter (Supplementary Figure S10), with values closer to 1 resulting in a behaviour that differs greatly from the observed in Figure 2d. This means that, in order to represent our measurements within TDGL, a different critical exponent is needed for the static and dynamical phase transitions.

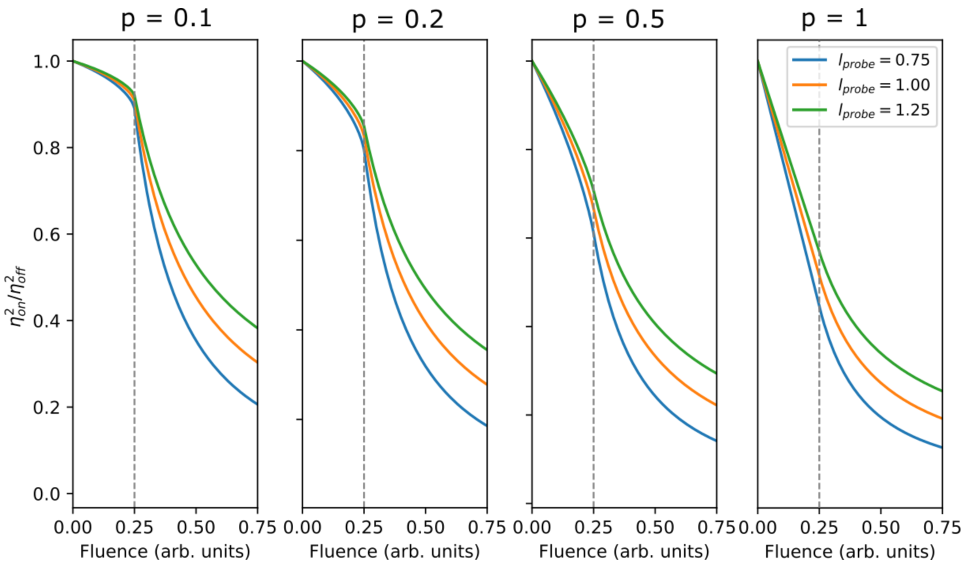

Supplementary Figure S9: Effect of the critical exponent on fluence dependence. For values of p around 0.2 the kink is always observed at the same fluence for any probe penetration length *l*. However, the contrast of the kink is progressively reduced as *p* is increased towards 1.

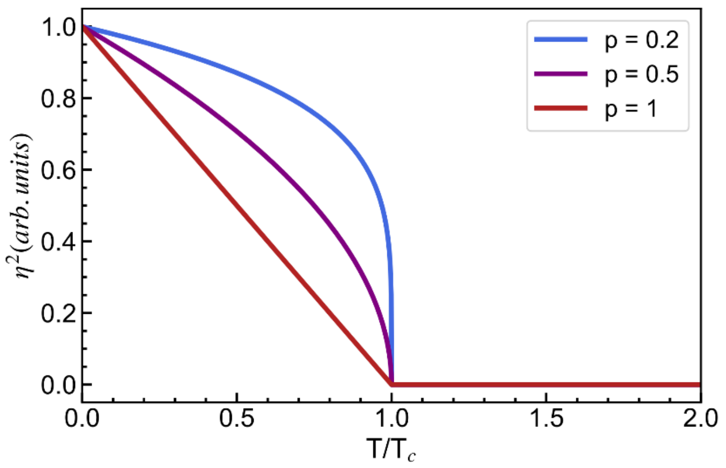

Supplementary Figure S10: Effect of the critical exponent on temperature dependence. The value of p affects the temperature dependence of the order parameter in a homogeneous second order transition.

Supplementary References

1. Johnson, A. S., Amuah, E. B., Brahms, C. & Wall, S. Measurement of 10 fs pulses across the entire Visible to Near-Infrared Spectral Range. *Sci. Rep.* **10**, 4690 (2020).
