# Supplementary material for: Multi-mode excitation drives disorder during the ultrafast melting of a C4-symmetry-broken phase
Source: Nat Commun. 2022 Jan 11;13:238. doi: 10.1038/s41467-021-27819-y (PMC8752725; doi:10.1038/s41467-021-27819-y)
Supplement: Supplementary file 3 — Description of Additional Supplementary Files [file 41467_2021_27819_MOESM3_ESM.docx]

Description of Additional Supplementary Files: Multi-mode excitation drives disorder during the ultrafast melting of a C4-symmetry-broken phase

**Daniel Perez-Salinas^1*^, Allan S. Johnson^1*^, D. Prabhakaran^2^, Simon Wall^1,3^**

*^1^ICFO – The Institute of Photonics Sciences, The Barcelona Institute of Science and Technology, 08860, Castelldefels, Barcelona, Spain*

*^2^Department of Physics, Clarendon Laboratory, University of Oxford, Oxford OX1 3PU, United Kingdom*

*^3^Department of Physics and Astronomy, Aarhus University, Ny Munkegade 120, 8000 Aarhus C, Denmark*

**Equal contribution*

**File Name: Supplementary Video 1.mp4**
Description: Left, time-resolved reflection anisotropy pattern measured at 1500 nm, following 1.5 mJcm^-2^. Right, corresponding normalized values for *r* and *η^2^* at each time delay

**File Name: Supplementary Video 2.mp4**
Description: Left, time-resolved reflection anisotropy pattern measured at 1500 nm, following 8 mJcm^-2^. Right, corresponding normalized values for *r* and *η^2^* at each time delay

**File Name: Supplementary Data.zip**
Description: Raw data used to obtain *r* and *η^2^* for the figures in the manuscript.
